# Involvement of NLRP3 and NLRC4 Inflammasome in Uropathogenic *E. coli* Mediated Urinary Tract Infections

**DOI:** 10.3389/fmicb.2019.02020

**Published:** 2019-09-03

**Authors:** Vivek Verma, Surbhi Gupta, Parveen Kumar, Sonal Yadav, Rakesh Singh Dhanda, Rajni Gaind, Renu Arora, Niels Frimodt-Møller, Manisha Yadav

**Affiliations:** ^1^Dr. B.R. Ambedkar Center for Biomedical Research, University of Delhi, New Delhi, India; ^2^Department of Urology, University of Alabama at Birmingham, Birmingham, AL, United States; ^3^Stem Cell Laboratory, Longboat Explorers AB, SMiLE Incubator, Lund, Sweden; ^4^Department of Microbiology, Vardhman Mahavir Medical College and Safdarjung Hospital, New Delhi, India; ^5^Department of Obstetrics and Gynecology, Vardhman Mahavir Medical College and Safdarjung Hospital, New Delhi, India; ^6^Department of Clinical Microbiology, Rigshospitalet, Copenhagen, Denmark

**Keywords:** inflammasomes, nod like receptors (NLR), *NLRP3*, *NLRC4*, uropathogenic *Escherichia coli* (UPEC), urinary tract infection (UTI)

## Abstract

**Background:**

Inflammatory response during urinary tract infection (UTI) is mediated by innate immune defense. Nod like receptors (NLRs) have been proposed to work simultaneously beside TLR pathways to mediate pro-inflammatory response and maintain tissue homeostasis. Some *in vitro* reports have showed the involvement of *NLRP3* inflammasome during uropathogenic *Escherichia coli* (UPEC) mediated UTI. So we have sought to determine the status of various inflammasomes and their components in UPEC mediated UTI.

**Methods:**

A total of 186 females experiencing the first episode of UTI were recruited for the study and forty were found to be positive for UPEC (≥10^5^ CFU/ml) in their urine (*N* = 40). Further, we analyzed the expression of *NLRP3*, *NLRC4*, *NAIP*, *AIM2*, *ASC*, *CASPASE-4*, and *CASPASE-1* gene at mRNA and protein level in the blood of UPEC confirmed study subjects through real time qPCR and immunoblotting. Healthy females (*N* = 40) visiting the OPD for health checkups, family planning advice and subjects undergoing routine medical examinations, were recruited as healthy control subjects. Pro-inflammatory cytokines (IL-6, IL-8, IFN-γ, TNF-α and MCP-1) were measured in the plasma of patients and controls through ELISA. For investigation of the involvement of *NLRC4* and *NLRP3* inflammasome, *in vitro* studies were performed using co-immunoprecipitation and confocal microscopy.

**Results:**

Most of the inflammatory regulators studied (i.e., *NLRP3, NAIP, NLRC4, ASC*, and *CASPASE-1*) were found to be up-regulated at both mRNA and protein levels in the UPEC infected UTI patients. Also, pro-inflammatory cytokines (IL-6, IL-8, IFN-γ, TNF-α, and MCP-1) were found to be up-regulated in the patients group. However, no significant difference was observed in the expression of *AIM2* and *CASPASE-4* genes at both mRNA and protein levels. Further, *in vitro* studies have shown the involvement of NLRC4 inflammasome in UPEC infected THP1 derived macrophages.

**Conclusion:**

Involvement of *NLRP3* and *NLRC4* inflammasomes in UPEC infected UTI is evident from our findings. This is the first report showing levels of inflammasome and its components in UTI patients suggesting a possible role during UPEC mediated UTI. We have also reported the involvement of *NLRC4* inflammasome for the first time during UTI infection.

## Introduction

Urinary tract infections (UTIs) are known to be the most common and prevalent infectious diseases associated with community and healthcare settings (Nicolle, 2005). It is mostly caused by bacterial infections (Foxman et al., 2000), whereas *Escherichia coli* (*E. coli*) is responsible for 80% of the cases of UTI in an ambulatory population (Stamm, 2002), designated as uropathogenic *E. coli* (UPEC). UPEC get accustomed to live within the urinary tract and bypass the host’s immune response through the process of biofilm formation and urothelial cell invasion (Mulvey et al., 2000). *E. coli* acts as the most common cause of infection leading to inflammation in the urinary bladder (Echols et al., 1999).

A substantial population of macrophages resides in the submucosa of the urinary tract, and more cells are recruited to these sites following infection (Engel et al., 2008). Upon activation, these macrophages produce crucial cytokines and chemokines that modulate the activity of these and other immune cells in the vicinity, which markedly influence the timing and intensity of inflammatory responses during UTIs (Duell et al., 2013; Symington et al., 2015). Excessive inflammation may lead to the chronic or secondary condition, which is further involved in tissue damage and disease severity to the host. Innate immune receptors act as the first line of defense against infectious microbes, continuously monitoring the extracellular milieu as well as subcellular compartments. These receptors can either be extracellular, such as some of the TLR and C-type lectin receptors (CLR), or intracellular, such as nucleotide oligomerization domain (NOD) like receptors (NLR), Retinoic-acid inducible gene (RIG-I)-like receptors (RLR) and AIM2 (absent in melanoma 2)-like receptors (ALR) (Verma et al., 2016). Cytosolic immune receptors not only act as PRRs which recognize PAMPs, but also sense signals derived from the host commonly known as damage associated molecular patterns (DAMPs). These DAMPs include intracellular molecules such as ATP and high mobility group box 1 (HMGB1) protein as well as proteins derived from extracellular matrix. These PRRs trigger a downstream signaling cascade in the presence of specific ligands, resulting in the activation of transcription machinery inducing the production and release of pro-inflammatory cytokines. These cytokines further regulate the switch between tissue homeostasis and the inflammatory state, aimed at the removal of pathogens thus restoring normal tissue function (Medzhitov, 2008). TLRs are a class of proteins which recognize pathogen derived conserved molecules, sensing endogenous danger signals and further activating immune cell response. TLRs are type I transmembrane glycoproteins which recognize PAMPS and various ligands, such as purified lipopolysaccharides (LPS), lipopeptides, or lipoteichoic acid leading to activation of signaling pathways associated with pro-inflammatory cytokines and inflammation. Due to absence of signal peptide, pro-IL-1β and pro-IL-18 requires cysteine protease caspase-1, which helps in cleavage and secretion of an active form of these cytokines via two signal mechanism. The first signal ‘priming’ is associated with TLR engagement and gives rise to gene transcription and pro-IL-1β accumulation. Second signal activates inflammasomes through nucleotide binding domain, leucine-rich repeat-containing proteins, i.e., NLRs which result in the activation of capase-1 and transformation of TLR mediated pro-IL-1β into mature IL-1β (van de Veerdonk and Netea, 2011). Due to expression at the cellular or endosomal membrane levels, TLR system does not recognize the intracellular bacterial communities (IBCs) and other intracellular pathogens, whereas a family of NOD proteins confirmed the presence of pathogens in the host cells (Schroder and Tschopp, 2010).

Inflammasomes are multiprotein complexes consisting mainly of NLR, ASC (apoptosis associated speck-like protein containing caspase recruitment domain [CARD]) and caspase-1, which are formed upon activation by specific ligands. NLRs are cytosolic protein receptors, and under highly regulated conditions they assemble with *ASC* and *CASPASE-1* to form speck-like aggregates (Jones and Dangl, 2006; Martinon et al., 2009). The composition of inflammasomes varies in response to different ligands, for example *NLRP1* is a sensor of MDP (muramyl dipeptide: toxin of *Bacillus anthracis*) (Boyden and Dietrich, 2006), *NLRP3* responds to various cytosolic insults like ROS (reactive oxygen species) (Cruz et al., 2007), cathepsin B release (Hornung et al., 2008), PFTs (pore forming toxins: nigericin, Hentze et al., 2003), extracellular crystals (Dostert et al., 2008), and *NLRC4* that oligomerizes in response to the presence of flagellin and PrgJ rod proteins within the cytoplasm (Zhao et al., 2011). *NLRC4* does not interact with PAMPs directly, rather a NLR family of proteins known as NAIPs (NLR family, apoptosis inhibitory proteins) recognize ligands for activation of *NLRC4* inflammasome (Schroder and Tschopp, 2010). Mouse genome carries different NAIPs for recognition of various microbial ligands, like NAIP1 (bacterial needle protein) (Yang et al., 2013), NAIP2 (PrgJ inner rod protein) and NAIP5/6 (bacterial flagellin) (Zhao et al., 2011), whereas in humans only one *NAIP* is known for recognition of flagellin and needle proteins (Yang et al., 2013). Pro-inflammatory response through cytokine (IL-1β and IL-18) release and pyroptosis are not the only functions of NLRC4 and NAIP axis. Their activation affects other aspects of cellular functions that play an important role in host defense. In a study, macrophages infected with *Salmonella* have shown antibacterial response through NLRC4 dependent actin polymerization, which prevents further bacterial uptake and increases intracellular ROS to enhance bacterial killing and decrease bacterial dissemination (Man et al., 2014). Epithelium specific deletion mutants of NAIP1-6 and NLRC4 resulted in higher bacterial load and extra intestinal dissemination during *Salmonella* infection, indicating their importance in regulating the bacterial load during disease (Sellin et al., 2014). In a mouse model study, NLRC4 dependent and cytokine (IL-1β and IL-18) independent mortality is seen in 30 min of intraperitoneal delivery of flagellin (von Moltke et al., 2012). This noticeable effect of Inflammasome was via increased production of eicosanoids (including prostaglandins and leukotrienes), leading to rapid vascular fluid loss and mortality (von Moltke et al., 2012).

In kidney nitric oxide (NO) regulates multiple processes, like fluid and salt reabsorption, renal hemodynamics, rennin secretion and tubuloglomerular feedback (Ortiz and Garvin, 2002). Inducible form of nitric oxide synthase (iNOS) is expressed during microbial infection and in response to inflammatory cytokines (Fang, 2004). Higher levels of NO or iNOS are evident in urine of the UTI patients (Lundberg et al., 1997), with localization of iNOS in neutrophils, kidney and bladder epithelial cells of murine animal models (Poljakovic et al., 2001; Choi et al., 2012). In some tissues, production of NO regulates secretion of chemokines and cytokines directly affecting the induction and resolution of inflammation (Kobayashi, 2010). Recently we have reported decreased levels of NO and increased levels of cytokine IL-1β in circulating blood plasma of UPEC infected UTI patients (Verma et al., 2018). Decreased levels of NO indicate high levels of free radicals, which in turn regulate the inflammasome pathway (Jo et al., 2016), thus in this study we intend to explore the status of inflammasome components in UPEC infected UTI patients.

To the best of our knowledge, no such report is available on inflammasome gene expression at mRNA and protein levels in UPEC infected UTI. In the current study, we have investigated the expression of various inflammasomes and levels of pro-inflammatory cytokines in UPEC infected UTI patients. In addition to this, we have studied the gene expression profile of *NLRP3*, *NAIP*, *NLRC4*, *AIM2*, *ASC*, *CASPASE-4*, and *CASPASE-1* at mRNA and protein levels in blood samples collected from UPEC infected UTI patients. Additionally, we have also checked the interaction of NLRP3, NLRC4, and NAIP with ASC after CFT073 (acute pyelonephritis strain of UPEC) infection under *in vitro* conditions. The present study is a step toward investigating the role of inflammasomes and pro-inflammatory markers in the pathogenesis of UTI.

## Materials and Methods

### Materials

SIGMAFAST^TM^ Protease Inhibitor Cocktail Tablets (Sigma-Aldrich Cat # S8830; United States), Bicinchoninic acid (BCA) assay kit (Merck Cat # 71285; India), PVDF membrane (GE Healthcare Life Sciences Cat# 10600023; United States), CFT073 (ATCC # 700928; United States), Luria-bertani broth (Himedia cat # M1245; India), Agar (Himedia cat # GRM666; India), THP-1 cells (ATCC # TIB-202; United States), RPMI-1640 (Gibco Cat# 11875119; United States), fetal bovine serum (Gibco Cat# 10270106; United States), Phorbol 12-myristate 13-acetate (PMA) (Sigma-Aldrich Cat# P1585; United States), anti-NLRP3 antibody (NovusBiologicals Cat#NBP1-77080; United States), anti-NLRC4 antibody (NovusBiologicals Cat# NBP2-41124; United States), anti-NAIP antibody (NovusBiologicals Cat # NBP1-77196; United States), anti-Caspase-1 antibody (NovusBiologicals Cat # NBP1-45433; United States), anti-ASC antibody (NovusBiologicals Cat # NBP1-78977; United States), anti-Caspase-4 (NovusBiologicals Cat # NBP1-77208; United States), anti-AIM2 (Abnova Cat # H00009447-B01P), anti β-actin (GeneTex Cat # GT5512) and anti-GAPDH antibody (Santa Cruz Cat # sc-47724; United States), Horseradish Peroxidase-labeled (HRP) anti-mouse (NovusBiologicalsCat # NB7539; United States) and anti-rabbit (Abcam Cat # ab6721; United States) secondary antibody, Clarity western ECL substrate (Bio-Rad Cat # 1705060; United States), Alexa Fluor 488 (Invitrogen Cat # A-11008; United States), Recombinant Protein A-Sepharose 4B (Invitrogen Cat # 101141; United States), VECTASHIELD Antifade Mounting Medium (Vector laboratories, Cat # H-1000; United States), TRI Reagent (Sigma Cat # T9424; United States) and RevertAid First Strand cDNA Synthesis Kit (Thermo Scientific Cat # K1622; United States) were used.

### Study Subjects

A total of 186 female patients (*N* = 186) were recruited from the outpatient clinics of Department of Obstetrics and Gynecology, Vardhman Mahavir Medical College (VMCC) and Safdarjung Hospital, New Delhi, India according to the following criteria: (1) Patients age: 18–55 years with gynecological problem. (2) Clinical history of burning micturition, frequent dysuria, abdominal pain, loin tenderness, dysfunctional voiding, hematuria and fever. (3) Patients with ≥10^5^ colony-forming units per milliliter (CFU/mL) of UPEC bacteria in urine. Patients with a history of severe allergic reactions, suspected/confirmed pregnancy, and malignancy of reproductive tract, suffering from metabolic diseases (hypertension, arthiritis, diabetes, Hypo/hyper thyroidism etc.), systemic diseases and immunocompromised state, were excluded from the study. Patients received/receiving any kind of antibiotic therapy, less than 14 days prior to enrolment, having problem of Urolithiasis, undergone any recent surgical procedure and recent catheterization, or known anatomic or functional abnormalities of the urinary tract were also excluded.

Forty (*N* = 40) healthy female subjects from similar socio-economical background age 18–55 years, who participated in voluntary health checkups, family planning advice and subjects undergoing routine medical examinations, were recruited from outpatient department as healthy control subjects. The study subjects having suspected/confirmed pregnancy, malignancy of reproductive tract, history of severe allergic reaction, suffering from metabolic diseases (hypertension, arthiritis, diabetes, Hypo/hyper thyroidism etc.) and any other simultaneous infections, systemic diseases, immunocompromised state, received/receiving any kind of antibiotic therapy, less than 14 days prior to enrolment, were excluded from the study. Controls having dysfunctional uterine bleeding, vaginal Polyp, problem of urolithiasis, recent catheterization, or known anatomic or functional abnormalities of the urinary tract or undergone any surgical procedure, were also excluded from the present study.

We have reported the expression of inflammasomes and its components at mRNA and protein levels for the first time in UPEC infected UTI patients. On the basis of previous studies regarding levels of inflammasomal mRNA expression reported in peripheral blood (Alcocer-Gómez et al., 2014; Satoh et al., 2014), the sample size was estimated using statistical software and sample size calculation methods, reported earlier (Cristofolini and Testoni, 2000; Charan and Biswas, 2013). Accordingly, 40 UTI patients and 40 controls were included to detect a statistically significant difference at two sided 5% α error and with 80% power of study.

### Urine and Blood Sample Processing

#### Urine Samples

Urine samples were obtained from all the study subjects and processed in the Department of Microbiology, VMMC and Safdarjung Hospital, New Delhi, India. Analysis of urine for various parameters such as, determination of specific gravity, pH, glucose, protein, blood, leukocyte esterase and nitrite was carried out by dipstick analysis (Ames-N; Miles-Sankyo, Tokyo, Japan) as per manufacturer’s protocols. Out of total 186 patients, 40 patients (21.5%) were confirmed urine culture positive for *E. coli* and 16 were positive (8.6%) for other microbes (*Staphylococcus aureus, klebsiella, Proteus, Entrococcus and Gram positive cocci*). Patients positive for only *E. coli* were included in the study. Therefore, a total of 80 study subjects, 40 UPEC infected UTI patients and 40 age matched healthy controls were included in the present study.

#### Blood Samples

Five milliliter (5 ml) of peripheral blood was collected from all the subjects who participated in this study in an ethylene diamine-tetra acetic acid (EDTA) vial. Three ml of whole blood was used for RNA and protein isolation. Remaining two ml of blood was centrifuged at 520 g for 10 min to separate plasma and cell pack volume, which was stored at −80°C for further use. Plasma was used for cytokine and nitrite estimation. Three ml of blood was subjected to red blood cell (RBC) lysis by using RBC lysis buffer (155 mM NH_4_Cl, 12 mM NaHCO_3_ and 0.1 mM EDTA) (Cold Spring Harbor Protocols, 2006). RBC lysis buffer was added and was subjected to invert mixing for 15 min on rotatory mixer at room temperature (RT) and there after centrifuged at 3000 *g* for 10 min at RT and supernatant was discarded. White pellet containing white blood cells (WBCs) was again dissolved in the same amount of RBC lysis buffer and this step was repeated twice to remove any RBCs left in the solution. Further WBCs were washed twice in 1X PBS (pH = 7.4) and dissolved in 1X PBS. The WBC solution was centrifuged at 3000 *g* for 10 min at 4°C. Supernatant was discarded and pellet was further processed to obtain RNA and protein.

### RNA Isolation and cDNA Synthesis

Total RNA from WBC pellet was extracted by using TRI reagent (Sigma) as per manufacturer’s instructions and quantified by using Nanodrop (ND-1000). Remaining red organic phase during RNA isolation was saved to isolate protein. A 1000 ng of total RNA after quantification was subjected to cDNA synthesis using cDNA synthesis kit [RevertAid First Strand cDNA Synthesis Kit (Thermo Scientific)] as per manufacturer’s protocol.

### Quantitative Real Time PCR

The gene expression levels of *NLRP3, NLRC4, NAIP, AIM2, ASC, CASPASE-4*, and *CASPASE-1* were determined by quantitative real time PCR for controls and UPEC infected UTI patients. ABI 7300 Real Time PCR machine was used for quantification of various genes by using Mesa green PCR Master mix (SYBR) (Eurogentec). The Real time qPCR reaction contained 7.5 μl of 2X Mesa green PCR Master mix, 1 μl cDNA, 1 μM of each primer and water to make final volume of 15 μl. qPCR conditions were: 50°C for 5 min, 95°C for 10 min, 40 cycles of 95°C for 15 s and 60°C for 1 min. To amplify the mentioned genes, primers were designed as shown in Table 1. Human *18S rRNA* was used as housekeeping gene for internal control (or reference gene) (Chandra et al., 2014). Human GAPDH gene was also amplified and analyzed in all samples in order to ensure the quality of cDNA (Supplementary Figure S1). Relative quantification values were expressed using the ΔΔCt method normalized to the reference gene and related to the expression of the controls (Ginzinger, 2002) and calculated as mentioned below. Normalization: delta-Ct (Patient) = (Ct of gene in Patient) – (Ct of 18S rRNA in Patient). delta-Ct (Control) = (Ct of gene in Control) – (Ct of 18S rRNA in Control). delta-delta-Ct = delta-Ct (Patients) – delta-Ct (Control). Relative quantification = 2^–(delta–delta–Ct)^.

**TABLE 1:** List of primers used in qRT-PCR for gene expression.

**S. No.**	**Gene**	**Accession no.**	**Primer sequence**
1	*NLRP3*	NM_004895.4	F: 5′-AGCCCCGTGAGTCCC ATTA-3′
			R: 5′-ACGCCCAGTCCAACATC ATCT-3′
2	*NLRC4*	NM_021209.4	F: 5′-TACACAGCAGGACGAAG ACTCAG-3′
			R: 5′-GGCTTCCACAGATGAC CCACA-3′
3	*NAIP*	NM_004536.2	F: 5′-TTACGAAGAACTACGGC TGGACT-3′
			R: 5′-GTCATCACCTTCCTGC CATTTC-3′
4	*CASPASE-1*	NM_001257118.2	F: 5′-TGAATACCAAGAACTG CCCAAG-3′
			R: 5′-GCATCATCCTCAAACTCTT CTGTAG-3′
5	*18S rRNA*(Chandra et al., 2014)	NM_022551.2	F: 5′-GTGGTGTTGAGGAAAGC AGACA-3′
			R: 5′-TGATCACACGTTCCACC TCATC-3′
6	*AIM2*	NM_004833.3	F: 5′-ATCTCCTGCTTGCCTTC TTGG-3′
			R: 5′-AAGTCTCTCCTCATGTTA AGCCTG-3′
7	*ASC*	NM_013258.5	F: 5′-AGTGGCTGCTGGATGC TCTG-3′
			R: 5′-CATCTTGCTTGGGTT GGTGG-3′
8	*CASPASE-4*	NM_001225.4	F: 5′- AAAGGAGAGAAACAACC GCACAC-3′
			R: 5′- TCGGAGGCAGATGG TCAAAC-3′
9	*GAPDH* (Précourt et al., 2012)	NM_002046.7	F: 5′-AGAAGGCTGGGGCT CATT-3′
			R: 5′- GGGCCATCCACAGT CTTCT-3′

### Bacterial Strain and Growth Conditions

*Escherichia coli* CFT073 (ATCC # 700928), a well-established acute pyelonephritis strain was used in this study, which was isolated from blood and urine culture of a woman. CFT073 was cultured at 37°C on solid or in liquid LB medium (48 h under static condition) (Palaniyandi et al., 2012) and OD^600^ was used to calculate MOI, which was used as per requirement of the experiment.

### Cell Culture

THP-1 cells (Cat # TIB-202, ATCC, Manassas, VA, United States) were cultured in RPMI-1640 medium supplemented with 10% heat inactivated fetal bovine serum, 2 mM L-glutamine, 1 mM sodium pyruvate and 10 mM HEPES (cat#15630080, Life Technologies, Carlsbad, CA, United States). THP-1 cells were differentiated into macrophage-like cells by culturing for 48 h in a medium containing 25 nM Phorbol 12-myristate 13-acetate (PMA) (Sigma-Aldrich, St Louis, MO, United States) and followed by rest of 24 h before any stimulation experiments (Lund et al., 2016).

### Stimulation Protocol

THP-1 derived macrophages (THP-1m) were incubated in RPMI 1640 medium with or without (Mock) CFT073 in an antibiotic free medium. THP-1m were stimulated by CFT073 for 6 h. CFT073 was used for infection at MOI (multiplicity of infection) of 1:5 for all experiments. THP-1m were stimulated by CFT073 for 6 hrs (used for all experiments).

### Lysate Preparation of THP-1m

After all incubation periods, cytoplasmic extract was prepared by lysing cells using cold Radioimmunoprecipitation assay (RIPA) lysis buffer (150 mM NaCl, 1% Nonidet P-40, 0.5% Sodium deoxycholate, 0.1% Sodium dodecyl sulfate and 25 mM Tris) (Cold Spring Harbor Protocols, 2017) (pH 7.4) supplemented with protease inhibitor cocktail for 15 min on ice. Homogenous lysis was achieved by passing cell suspension through 28 gauge needle syringe and then lysates were cleared by centrifugation at 13,000 rpm for 20 min, supernatant was collected as cytoplasmic extract. Cytoplasmic extract was stored at −80°C until required for further use.

### Confocal Microscopy

The expression of NLRC4 and NAIP was checked in THP-1m upon CFT073 infection by confocal microscopy. PMA treated THP-1 cells were seeded in 8 well chamber slides, washed with RPMI 1640 medium and followed by infection with CFT073 at 1:5 MOI for 6 hrs. After 6 h cells were rinsed with 1X PBS for 5 min and then fixed in 4% paraformaldehyde (pH 7.4) at 37°C for 30 min. Cells were permeablized with 0.15% of Triton X100 in 1X PBS for 10 min at room temperature (RT). Cells were washed thrice with 1X PBS followed by blocking with 1% BSA in PBST at RT for 30 min. Cells were incubated with anti-NLRC4 and NAIP antibody at 20 μg/ml concentration (Cat# NBP1-77080, Novus Biologicals) with 1% BSA in 1X PBS overnight at 4°C in dark. Thereafter, cells were washed with 1X PBS for 5 min and incubated with secondary antibody alexa fluor 594 at 2 μg/ml concentration (Cat# A-11012, Thermo Fisher Scientific) in 1% BSA for 2 h at RT in dark. Further, cells were washed thrice with 1X PBS for 5 min and incubated with DAPI at 300 nM concentration in 1X PBS for 5 min followed by washing in 1X PBS. Cells were mounted with VECTASHIELD Antifade Mounting Medium (Cat# H-1000, Vector Laboratories). Confocal imaging was performed with a Nikon A1 laser scan confocal microscope with Plan Apo optics equipped with an argon laser. Data were analyzed using the NIS Elements Advanced Research software. Each experiment was performed three times in duplicates.

### Co-immunoprecipitation

Interaction and oligomerization of ASC with NLRP3, NLRC4 and NAIP upon CFT073 infection was checked by standard co-immunoprecipitation method. In brief, cytoplasmic extract of THP-1m after all stimulation protocols were stored at −80°C for further use. Five hundred μg of protein from cytoplasmic extracts were thawed in ice and incubated with 1 μg of anti-ASC (NovusBiologicals) antibody and 20 μl of recombinant Protein A-Sepharose 4B beads (Invitrogen) for overnight at 4°C on a rotary invert mixer. Next day Sepharose beads were washed thrice with RIPA lysis buffer and 2X SDS PAGE protein sample buffer (80 mM TrisHCl (pH 6.8), 10% (v/v) Glycerol, 2% SDS, 238 mM β-Mercaptoethanol, 0.0006% (v/v) Bromophenol blue and 0.1 M dithiothreitol [freshly added]) (Cold Spring Harbor Protocols, 2013) was added and boiled at 95°C for 5 min. For immunoblotting boiled samples were loaded and run on 8% resolving SDS-PAGE. Each experiment was performed three times in duplicates.

### Immunoblotting

The remaining red organic phase during RNA isolation was used to precipitate protein for immunoblotting according to the manufacturer’s protocol (TRI Reagent, Sigma). Protein was stored at −80°C and further for protein estimation and immunoblotting. Protein estimation of cytoplasmic extract was done using a BCA kit (Merck) according to the manufacturer’s instructions. Further, 50 μg of total protein was run on 15 and 8% (as required to detect desired molecular weight of protein) SDS-PAGE and subsequently transferred onto PVDF membrane (GE Healthcare Life Sciences) using 25 volts overnight at 4°C. The various protein molecules were probed with specific primary antibodies (NLRP3, NLRC4, NAIP, AIM2, ASC, Caspase-4, and Caspase-1), followed by HRP-labeled anti mouse and anti rabbit secondary antibody. The Clarity western ECL substrate (Bio-Rad) was used to develop the blot by chemiluminescence. Quantification was carried out using ImageJ software (NIH). GAPDH and β-actin were used as internal control in immunoblotting.

### Cytokine Measurement

Plasma separated from the blood (UTI patients and control group) sample was used for measurement of different cytokine levels. Cytokines IL-6, IL-8, IFN-γ, TNF-α and MCP-1 were estimated by enzyme-linked immunosorbent assay (ELISA) using Ready-SET-Go ELISA kits (eBiosciences, San Diego, CA, United States). ELISA was performed strictly as per the manufacturer’s instructions.

## Results

### Gene Expression Analysis of *NLRP3, NLRC4, NAIP, AIM2, ASC, CASPASE-4*, and *CASPASE-1*

**FIGURE 1:**
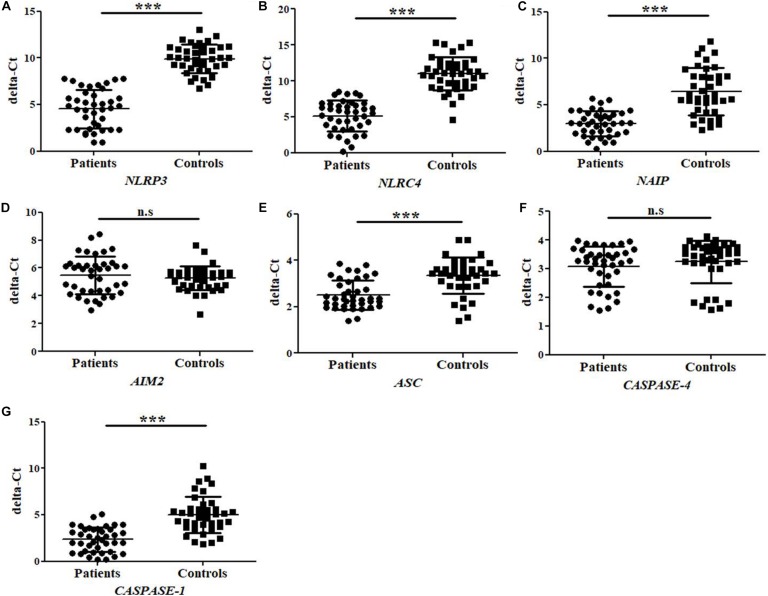
Bar Graphs representing delta-Ct values for study subjects (patients and controls) regarding **(A–G)** *NLRP3, NLRC4, NAIP, AIM2, ASC, CASPASE-4*, and *CASPASE-1* gene expression. The higher delta-Ct value represents the lower expression of gene at mRNA level. Values are shown as Mean ± SD. *P*-value is ^∗∗∗^*p* = 0.0001 and n.s, non-significant.

The mRNA expression for different inflammasomes and its components such as, *NLRP3, NLRC4, NAIP, AIM2, ASC, CASPASE-4*, and *CASPASE-1* genes were performed by quantitative Real time PCR in UPEC infected UTI patients (*N* = 40) and in controls healthy group (*N* = 40). The average delta-Ct value for *NLRP3*, *NLRC4, ASC*, *NAIP*, and *CASPASE-1* genes were significantly (*p* = 0.0001) lower in patients (4.5 ± 2.1, 5.1 ± 2.1, 2.5 ± 0.6, 3.0 ± 1.3, and 2.4 ± 1.3) as compared to controls group (9.9 ± 1.5, 11.0 ± 2.3, 3.3 ± 0.8, 6.4 ± 2.5, and 5.0 ± 1.9), respectively (Figure 1 and Table 2). However, the average delta-Ct value for *AIM2* and *CASPASE-4* genes were not significantly different (*p* = 0.4 and *p* = 0.3) among patients group (5.5 ± 1.4 and 3.1 ± 0.7) as compared to controls (5.3 ± 0.13 and 3.3 ± 0.8), respectively (Figure 1 and Table 2). The delta-delta-Ct value for *NLRP3, NLRC4, NAIP, AIM2, ASC, CASPASE-4*, and *CASPASE-1* in patients was observed to be −5.4, −5.9, −3.4, 0.2, −0.8 and −2.6 respectively (Table 2).

**TABLE 2:** Table showing data of gene expression at mRNA level of *NLRP3*, *NLRC4, NAIP, CASPASE-1, AIM2, ASC*, and *CASPASE-4.*

**Genes**	**Avg. delta-Ct ± SD Patients *N* = 40**	**Avg. delta-Ct ± SD Controls *N* = 40**	**delta-delta-Ct**	**Fold change**	***P*-value**
*NLRP3*	4.5 ± 2.1	9.9 ± 1.5	−5.4	41.3	^∗^*P* < 0.0001
*NLRC4*	5.1 ± 2.1	11.0 ± 2.3	−5.9	57.9	^∗^*P* < 0.0001
*NAIP*	3.0 ± 1.3	6.4 ± 2.5	−3.4	10.8	^∗^*P* < 0.0001
*CASPASE-1*	2.4 ± 1.3	5.0 ± 1.9	−2.6	6.3	^∗^*P* < 0.0001
*AIM2*	5.5 ± 1.4	5.3 ± 0.8	0.2	0.8	*P* = 0.3986
*ASC*	2.5 ± 0.6	3.3 ± 0.8	−0.8	1.8	^∗^*P* < 0.0001
*CASPASE-4*	3.1 ± 0.7	3.2 ± 0.7	−0.1	1.1	*P* = 0.3094

*delta-Ct, delta-cycle threshold; delta-delta-Ct, delta-delta-cycle threshold. Expression at mRNA levels of *NLRP3*, *NLRC4, NAIP, ASC*, and *CASPASE-1* were increased in UTI patients as compared to controls. Patients group was compared with controls. ^∗^*P* < 0.05 is considered to be significant.*

The negative values of delta-delta-Ct in patients indicate upregulation of *NLRP3, NLRC4, NAIP, ASC, CASPASE-1*, and *CASPASE-4* genes. However, positive delta-delta-Ct value refers to the down-regulation of *AIM2* mRNA expression in the patients group. We have observed average fold change of 41.3, 57.9, 10.8, 0.8, 1.8, 1.1, and 6.3 folds in mRNA expression levels of *NLRP3, NLRC4, NAIP, AIM2, ASC, CASPASE-4*, and *CASPASE-1* genes respectively in patients as compared to control group (Table 2). We may conclude that *NLRP3, NLRC4, NAIP, ASC*, and *CASPASE-1* genes were observed to be upregulated whereas expression analysis of *AIM2* and *CASPASE-4* showed no statistical difference, between patients and control group.

### Protein Expression Analysis of NLRP3, NLRC4, NAIP, and Caspase-1

**FIGURE 2:**
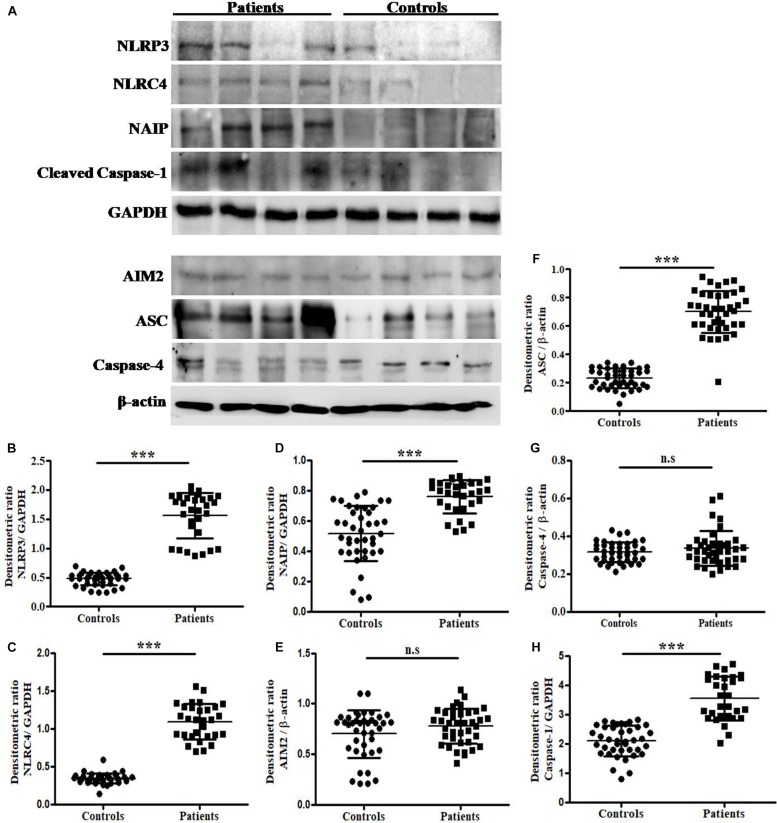
Analysis of NLRP3, NLRC4, NAIP, AIM2, ASC, Caspase-4, Caspase-1, β-actin and GAPDH protein expression by immunoblotting. **(A)** Represents the immunoblotting results of NLRP3, NLRC4, NAIP, Caspase-1, GAPDH, AIM2, ASC, Caspase-4 and β-actinin UPEC infected UTI patients and controls group. Scattered plots showing individual densitometric values (IDV) of NLRP3 **(B)**, NLRC4 **(C)**, NAIP **(D)**, AIM2 **(E)**, ASC **(F)**, Caspase-4 **(G),** and Caspase-1 **(H)**. Results were expressed as average densitometric ratio in patients and controls group ± SD. *P*-value is ^∗∗∗^*p* = 0.0001 and n.s, non-significant.

The total protein extracted by trizol method was subjected to immunoblotting and expressed in terms of integrated densitometric value (IDV) for individual inflammasome and its components in UTI patients and in controls group (Figure 2A). The observed IDV for NLRP3, NLRC4, NAIP, ASC and Caspase-1 protein was significantly (*p* = 0.0001) higher in patients group (1.56 ± 0.39, 1.10 ± 0.23, 0.76 ± 0.11, 0.70 ± 0.15, and 3.54 ± 0.75) as compared to controls (0.48 ± 0.11, 0.35 ± 0.07, 0.52 ± 0.18, 0.23 ± 0.07, and 2.10 ± 0.0.52), respectively (Figures 2B–D,F,H). However, no significant difference (*p* = 0.09 and *p* = 0.24) was observed for AIM2 and Caspase-4 protein IDV values in patients group (0.78 ± 0.15 and 0.36 ± 0.09) as compared to controls (0.70 ± 0.24 and 0.36 ± 0.05), respectively (Figures 2E,G). Results were expressed as average densitometric ratio in patients and controls group ± SD.

### Levels of Pro-inflammatory and Immune-Regulatory Cytokines in the Patients and Control Study Subjects

**FIGURE 3:**
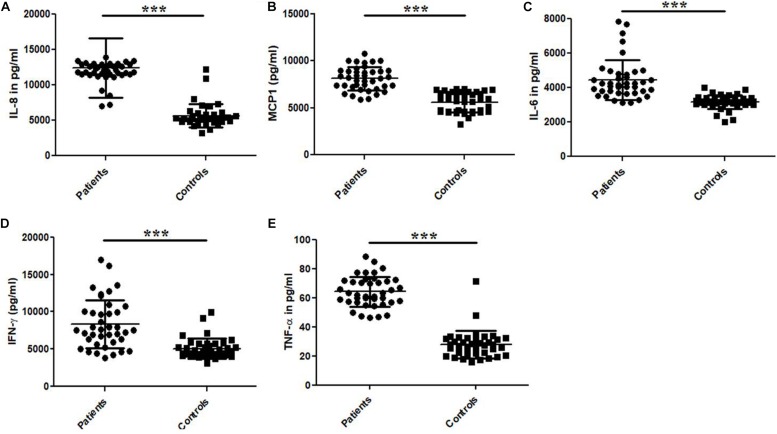
Bar graphs representing plasma levels of pro-inflammatory and immune-regulatory cytokines. **(A)** IL-8, **(B)** MCP-1, **(C)** IL-6, **(D)** IFN-γ, and **(E)** TNF-α in the UTI patients and controls group. Data is represented in terms of Mean ± SD in pg/ml. *P*-value is ^∗∗∗^*p* = 0.0001.

The cytokine (IL-6, IL-8, IFN-γ, MCP-1, and TNF-α) levels were measured by ELISA in the plasma samples collected from UPEC infected UTI patients and control study subjects (Figure 3 and Table 3). The cytokine levels for IL-6, IL-8, IFN-γ, MCP-1 and TNF-α were significantly (*p* = 0.0001) different in the patients and controls group and expressed in terms of Mean ± SD (Figure 3 and Table 3). The observed levels of IL-6 (4454 ± 1153), IL-8 (12459 ± 4193), IFN-γ (8346 ± 3235), MCP-1 (8119 ± 1236) and TNF-α (64.4 ± 10.4) in patients group was found to be significantly higher as compared to the controls group, i.e., IL-6 (3160 ± 399.5), IL-8 (5651 ± 1666), IFN-γ (5050 ± 1346), MCP-1 (5604 ± 1044), and TNF-α (27.9 ± 9.5), respectively (Figure 3 and Table 3).

**TABLE 3:** Comparison of the plasma cytokine levels between UPEC infected UTI patients and healthy controls.

**Study subjects**	**IL-8 (pg/ml)**	**IL-6 (pg/ml)**	**IFN-γ (pg/ml)**	**MCP-1 (pg/ml)**	**TNF-α**
Patients (*n* = 40)	12459 ± 4193	4454 ± 1153	8346 ± 3235	8119 ± 1236	64.4 ±c 10.4
Controls (*n* = 40)	5651 ± 1666	3160 ± 399.5	5050 ± 1346	5604 ± 1044	27.9 ± 9.54
*P*-values	*p* < 0.0001	*p* < 0.0001	*p* < 0.0001	*p* < 0.0001	*p* < 0.0001

*Values are expressed in terms of Mean ± SD. *P-*value for differences between cytokines of patients and control is statistically significant by unpaired *t*-test.*

### NLRP3, NLRC4, and NAIP Interact With ASC During CFT073 Challenge to THP-1m

**FIGURE 4:**
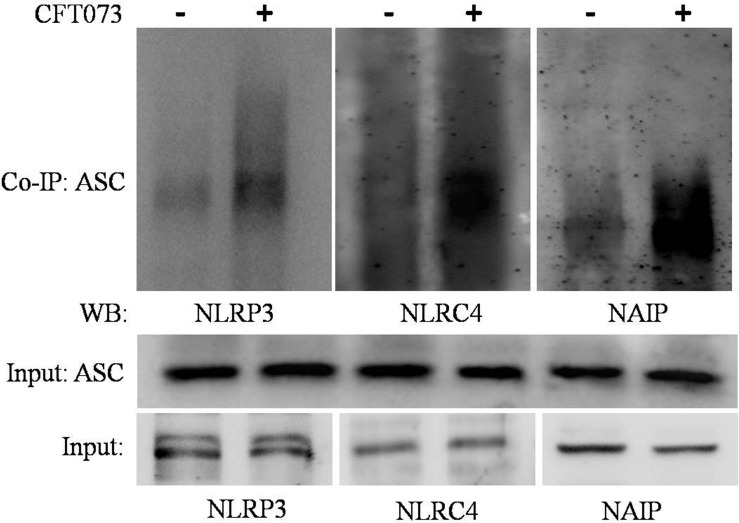
Co-immunoprecipitation assay: Figure showing interaction of NLRP3, NLRC4 and NAIP with ASC during CFT073 infection in THP-1m. Cytoplasmic extracts of THP-1m infected with or without CFT073, 6 h post stimulation were subjected to IP and WB by various antibodies (ASC, NLRP3, NLRC4, and NAIP) as described in material and methods. Cytoplasmic extract used as input was subjected to check NLRP3, ASC, NLRC4 and NAIP expression as shown in figure. ASC antibody was used to co-immunoprecipitate the complex from cytoplasmic extracts and then immunoblotted to detect NLRP3, NLRC4 and NAIP.

To confirm association of NLRP3, NLRC4 and NAIP with ASC during CFT073 infection of THP-1m, we performed immunoprecipitation of ASC and detected NLRP3, NLRC4 and NAIP proteins. Endogenous levels of NLRP3, ASC, NLRC4 and NAIP were immunoblotted in input lysates obtained from resting and CFT073 stimulated macrophages (Figure 4). During CFT073 infection, interaction of endogenous NLRP3, NLRC4 and NAIP with ASC was confirmed as shown in Figure 4. Whereas in resting THP-1m interaction of NLRP3, NLRC4 and NAIP with ASC was negligible.

### NLRC4 and NAIP Is Upregulated Upon CFT073 Infection in THP-1m

**FIGURE 5:**
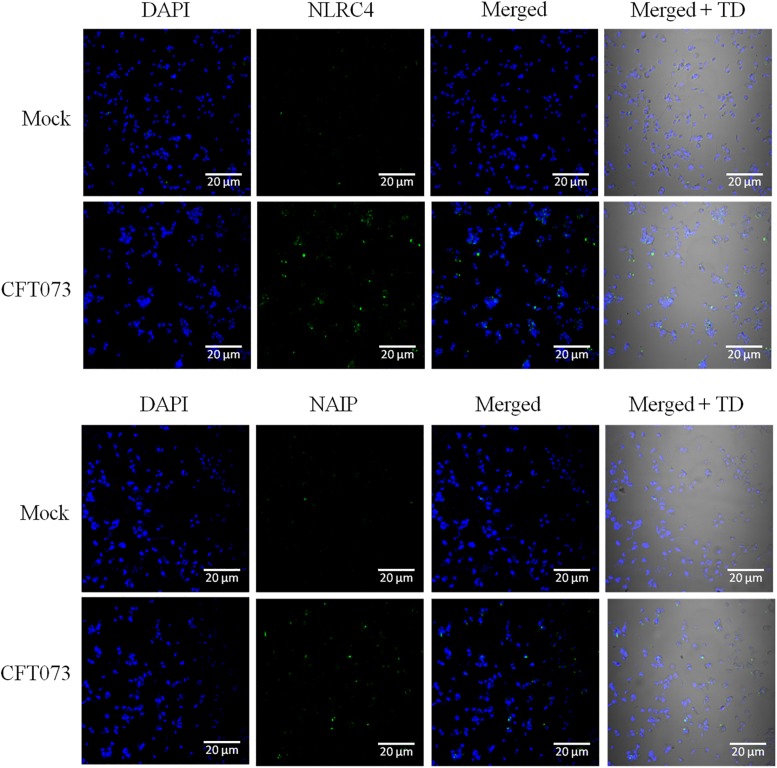
Confocal microscopy analysis of NLRC4 and NAIP expression in THP-1m with or without CFT073 stimulation. NLRC4 and NAIP expression in THP-1m, 6 h post stimulation with or without (mock) CFT073 was examined with confocal microscopy analysis using anti-NLRC4 and anti-NAIP antibody. Alexa fluor 488 (green) was used to detect anti-NLRC4 and anti-NAIP antibody. Nucleus of cells were stained with DAPI (blue). Results are representative of three independent experiments.

As it is evident from available literature that CFT073 carries ligand for NLRC4 inflammasome, i.e., flagellin, and best to our knowledge so far no study reported the involvement or a hint about the status of these inflammasomes during UPEC caused UTI. We tried to check the endogenous levels of NAIP and NLRC4 in an *in vitro* model where CFT073 stimulated THP-1m were subjected to confocal microscopy for comparing their status at resting (mock) and stimulated state. A clear upregulation of NLRC4 and NAIP in THP-1m upon CFT073 infection was seen as compared to resting macrophages (mock) (Figure 5).

## Discussion

UTI is one of the most common human infections which affects 50% of women once in their lifetime (Foxman, 2003). UPEC consists of various virulence factors, proteins and associated toxins, such as flagella, fimbrial adhesions, invasins and auto transported proteins (Ulett et al., 2013) which help them to attack the host cells and strongly entrench the infection. In response to infection, inflammation and tissue injuries occur (Medzhitov, 2008). However secondary damage and alteration in immune functions also occur due to dysregulated and chronic inflammation (Cirl et al., 2008; Yadav et al., 2010).

In context with UTI, pro-inflammatory cytokines and their increased production are closely related with UPEC infections in human subjects. Bacterial infection introduces the secretion of IL-6 and IL-8 (Hedges et al., 1991; Andersson et al., 1992). A previous study revealed that *E. coli* activated the recurrent secretion of cytokines, including IL-6 and IL-8 (Agace et al., 1993), play an indispensable role in mucosal immune system. Recent studies in human cell lines depicted that *E. coli* induced a significant increase in cytokine levels (IL-1 family, IL-6 and IL-8) (Brauner et al., 2001) and mRNA expression of IL-6 and chemokine MCP-1 up to four times, respectively (Nebel et al., 2013). We observed that plasma levels of pro-inflammatory and immune-regulatory cytokines such as IL-6, IL-8, IFN-γ, MCP-1 and TNF-α were significantly (*p* = 0.0001) higher in UPEC infected UTI patients as compared to healthy control (Figure 3 and Table 3). The elevated levels of cytokines in association with UPEC were reported in various studies (Hedges et al., 1991; Andersson et al., 1992; Agace et al., 1993; Brauner et al., 2001; Nebel et al., 2013), which supported our findings. The up-regulated levels of these cytokines in UPEC infected UTI patients may provide the diagnostic basis for infections and the involvement of inflammatory response for the bacterial clearance. The release of various pro-inflammatory cytokines triggers the immunological response together with the inflammasomal activation by DAMP, which plays a major role in UTI. Thus the cytokine profiling and their up-regulated levels in UTI patients may be important for the evaluation of possible infection and could be used as a better diagnostic tool for disease pathogenesis (Agace et al., 1993).

Hughes et al. (2016) reported that NLRP3 inflammasome had an important role in the induction and progression of inflammation, bladder outlet obstruction and bladder dysfunction in female mice (Hughes et al., 2016). They further showed that inhibition of inflammasomal activation (NLRP3) may directly influence the levels of IL-1β and IL-18 secretion and significantly reduce the bladder inflammation in various urological conditions (Hughes et al., 2016). It might be due to the arrest of functional changes related with bladder inflammation (voiding frequency and volume), which were induced by NLRP3 inflammasome. In an earlier preliminary study from our laboratory in UPEC infected UTI patients, we have observed decreased levels of NO and increased levels of cytokine IL-1β (Supplementary Figure S2) in circulating blood plasma (Verma et al., 2018). Decreased levels of NO indicate high levels of free radicals, which in turn regulates the inflammasome pathway (Jo et al., 2016), thus in the present study we intend to explore the status of inflammasome components in UPEC infected UTI patients.

Expression analysis for various inflammasomes in animal model suggested that NLRP1, NLRP3, and AIM2 were highly expressed in Kupffer cells and liver sinusoidal endothelial cells, while virtually absent in primary cultured hepatocytes in inflammatory and hepatic disease conditions (Boaru et al., 2012). Inflammatory genes, genetic variants and their expression are well established in several diseases. *NLRP3* has been associated with the development of cancer, atherosclerosis (Varghese et al., 2016), currently viewed as predictor for chronic myeloid leukemia (Zhang et al., 2018) and novel diagnostic biomarker for early detection for diabetic nephropathy (El-Horany et al., 2017). Another study in glomerular disease also suggested the elevated mRNA levels of *NLRP3* and *CASPASE-1* gene with respect to normal individuals (Xiong et al., 2015). We have recently reported the role of different inflammasomes and their signaling mechanism in patients with sexually transmitted disease (Verma et al., 2016). A recent study suggested that expression of *NLRP3* and *ASC* were elevated at mRNA and protein levels in blood samples of patients with Behçet’s disease as compared to healthy subjects (Kim et al., 2015). It is evident from available literature that mRNA and protein levels of *NLRP3* and *CASPASE-1* are upregulated in inflammatory pathologies of various diseases (Choulaki et al., 2015; Niu et al., 2015; Xiong et al., 2015).

Analysis of the expression of various inflammasomes, in the present study also showed a significant difference in the average delta-CT value of *NLRP3*, *NLRC4*, *NAIP, ASC*, and *CASPASE-1* gene expression between patients and controls group (Figure 1 and Table 2). In our study, we observed that the expression of *NAIP, ASC*, and *CASPASE-1* genes was significantly upregulated upto 10.8, 1.7, and 6.3 folds in patients in comparison to normal controls, respectively (Table 2). Similarly, for *NLRP3* and *NLRC4*, gene expression was highly upregulated and about 41.3 and 58 fold in patients group as compared to controls (Table 2).

Most of the *E. coli* isolated from UTI patients contains an important pore-forming toxin commonly known as α-hemolysin (HlyA) (Hannan et al., 2008). Expression of HlyA in UPEC infection mainly associated with urothelial cell toxicity and increased urothelial damage, reported in *in vitro* (Mobley et al., 1990) and *in vivo* studies (Smith et al., 2008). HlyA is associated with lipoproteins and has been reported to activate caspase-1 through inflammasomal activation in mice and human monocytic cells (Nagamatsu et al., 2015). The over expression of HlyA promotes NLRP3-dependent urothelial cell death, subsequently repressed in the presence of caspase inhibitors (Nagamatsu et al., 2015). Caspase-1 mRNA is translated into procaspase-1 thereafter on stimulation and inflammasome oligomerization, it is converted into a cleaved form of caspase-1 through autocatalytic cleavage (Schroder and Tschopp, 2010). On further analysis, we observed that expression of these inflammasomes at protein levels was significantly elevated (*p* = 0.0001) in UTI patients as compared to healthy controls group (Figure 2). Our results revealed that expression of NLRP3, ASC and cleaved Caspase-1 protein was increased upto 1.5-3 folds in patients as compared to controls group (Figure 2). Similarly, NAIP and NLRC4 protein expression levels were also up-regulated in patient group as compared to controls (Figure 2). The role of NLRP3 in inflammatory conditions has already been established, which is associated with its increased gene expression (Mitroulis et al., 2010). Fougeray and Pallet (2015) reported that expression of inflammasomes and their components may change at different time intervals, particularly in immunogenic cells, tubular epithelium and podocytes of upper urinary tract (Fougeray and Pallet, 2015). A recent study in murine UTI model revealed that CFT073 infection subsequently induced the expression of NLRP3 inflammasome, however the presence of TcpC may alter the release of mature IL-1β resulting in decreased levels of IL-1β (Waldhuber et al., 2016). An up-regulated expression of NLRP3 in glomeruli, might be correlated to the presence of disease conditions such as glomerulosclerosis or other age-related renal disorders (Fougeray and Pallet, 2015). Animal studies in murine UTI model revealed that CFT073 infection promptly induced NLRP3 expression in bladder mucosa (Waldhuber et al., 2016) and NLRP3 mRNA expression was significantly increased at early points after wild type UPEC infection (Nagamatsu et al., 2015), in consistent with our results. We have observed higher levels of caspase-1 expression in UPEC infected UTI patients at mRNA (Figure 1) as well as protein level (Figure 2). The expression levels of pro and cleaved form of caspase-1 were highly upregulated in patients as compared to control group (Figure 2 and Supplementary Figure S3). This further explains the role of autocatalytic cleavage of caspase-1 protein, acting as a checkpoint for release of pro-inflammatory cytokines (IL-1β and IL-18). In agreement with our findings, it has been reported that UPEC strains induced processing of procaspase-1 in human and mouse macrophages (Schaale et al., 2016). Cleavage of procaspase-1 into caspase-1 is NLRP3 dependent during UPEC infection (Schaale et al., 2016). Waldhuber et al. (2016) had also reported the cleavage of caspase-1 during CFT073 challenge to the human bladder epithelial cells and murine macrophages.

As UPEC carries ligands (Ulett et al., 2013) for NAIP and NLRC4 in its arsenal/virulence factors, therefore, we focused to check whether their expression is also affected in UPEC infected UTI or not. We observed upregulated levels at mRNA and protein levels in UTI patients as compared to controls (Figures 1, 2 and Table 2). Further, verification for upregulation of NLRC4 and NAIP was confirmed by *in vitro* stimulation of THP-1m by CFT073. In our study, confocal microscopy revealed upregulation of NLRC4 and NAIP in CFT073 infected THP-1m cells as compared to mock (Figure 5). In our study, the upregulated levels of *NAIP* gene expression may provide the basis for activation of the *NLRC4* and their inflammatory response, which is further shown by increased fold change expression of *NLRC4* at mRNA and protein levels in UPEC infected UTI patients. Previous biochemical studies already discussed that NAIP family of NLR proteins are known for their function toward bacterial ligands recognition and further trigger the activation of NLRC4 inflammasome (Zhao and Shao, 2015). UPEC carries flagellin and type 3 secretion system for their survival and host invasion, which can efficiently activate NLRC4 inflammasome. NAIP5 and NAIP6 recognize cytosolic presence of flagellin, whereas NAIP1 and NAIP2 recognize bacterial needle and inner rod proteins of the T3SS, respectively (Kofoed and Vance, 2011; Zhao et al., 2011). Rod proteins from T3SS apparatus, share a similar secondary structure to the D0 domain of flagellin, which is responsible for activation of NLRC4 (Lightfield et al., 2008; Kofoed and Vance, 2011). In our study, we have found NLRC4 activation in response to CFT073 infection and thus we may conclude that this activation could be due to the presence of flagellin and T3SS of CFT073 (Figure 4). The binding of ligand is an important function for the co-oligomerization of NAIPs with the NLR family, including NLRC4, which explains the molecular basis for the detection of ligands and formation of inflammasome complex (Tenthorey et al., 2014). Zhao et al. (2016) showed that knockout mice for NAIP gene, were deficient to detect the bacterial flagellin, provide the information regarding genetic function, antibacterial defense mechanism and inflammatory response of NAIP.

In our study, upregulated levels of *NLRC4* expression might be associated to the presence of flagellin as a virulence factor in UPEC strains (Wright et al., 2005), which provides the ability to proceed in human urinary tract. Schaale et al. (2016) investigated the role of NLRP3 inflammasome and its components in human and mouse macrophages using CFT073 and UTI89 strains. Their findings suggested that NLRP3 was not solely responsible for the secretion of IL-1β, although they had excluded the NLRC4 inflammasome from their study. The complete mechanism of expression of NLRC4 inflammasome is still unclear. The levels of *NLRC4* gene expression in UPEC infected UTI patients were not studied so far. Additionally, we have also observed interaction through co-immunoprecipitation of endogenous ASC with NLRP3, NLRC4 and NAIP during CFT073 challenge to THP-1m cells (Figure 4).

The findings of this study supported the role of inflammasomes such as *NLRP3*, *NAIP*, *NLRC4*, *ASC* and *CASPASE-1* in the development of UTI. To the best of our knowledge, this is the first study wherein *NLRP3*, *NAIP*, *NLRC4*, *ASC* and *CASPASE-1* gene expression at mRNA and protein levels has been compared in blood samples from UPEC infected UTI patients. An increase in gene expression, protein expression and elevated levels of pro-inflammatory cytokines in UTI patients group may be considered as a factor for progression of UTI. The major limitation of present study is small sample size. Our observations are exploratory; an extended sample-sized patient follow-up study is warranted to ascertain the role of inflammasome and their expression as a risk factor for UTI and to understand the patho-physiological role of *NLRP3*, *NAIP*, *NLRC4*, and *CASPASE-1* gene in UTI.

An up-regulated levels of NLRP3, NAIP, NLRC4, ASC and caspase-1 gene expression at mRNA and protein levels and an up-regulation in the pro-inflammatory cytokines (IL-6, IL-8, IFN-γ, MCP-1, and TNF-α) levels were found in UPEC infected UTI patients and could be a key factor for the development of this disease. This is the first report regarding levels of inflammasome and its components in UTI patients suggesting possible role during UPEC infected UTI. Further studies in extended sample size are needed to understand the patho-physiological role of NLRP3, ASC, NAIP, NLRC4 and caspase-1 gene in UTI, and by further studies we could also use the vigor of expression of these or more genes related to this mechanism in order to differentiate between different stages of UTI. We have also reported first time the involvement of NLRC4 inflammasome during UPEC infected UTI. In chronic UTI these effectors could be targeted to evade the acute inflammatory response.

## Data Availability

The raw data supporting the conclusions of this manuscript will be made available by the authors, without undue reservation, to any qualified researcher.

## Ethics Statement

This study was conducted in accordance with the Declaration of Helsinki 1975 (and as revised in 1983 and later amendments) and an approval of Institutional Human Ethics Committee of VMCC and Safdarjung Hospital, New Delhi (No. IEC/SJH/VMMC/Project/Sept-14/490) and Dr. B.R. Ambedkar Center for Biomedical Research, University of Delhi, Delhi (No. F50-2/Eth. Com/ACBR/15) was obtained prior to the study. The study subjects were recruited on the basis of a standard questionnaire with inclusion and exclusion criteria. A written informed consent was obtained from each study subjects prior to participation in the study.

## Author Contributions

VV, SG, PK, RD, and MY designed the experiments and analyzed the data. VV, SG, PK, and SY performed the experiments. RD, NF-M, and MY conceptualized and directed the study, and wrote the manuscript. RA and RG contributed in enrollment of study subjects.

## Conflict of Interest Statement

The authors declare that the research was conducted in the absence of any commercial or financial relationships that could be construed as a potential conflict of interest.
