# Supplementary material for: Involvement of NLRP3 and NLRC4 Inflammasome in Uropathogenic E. coli Mediated Urinary Tract Infections
Source: Front Microbiol. 2019 Sep 3;10:2020. doi: 10.3389/fmicb.2019.02020 (PMC6734172; doi:10.3389/fmicb.2019.02020)
Supplement: Supplementary file 1 [file Data_Sheet_1.docx]

**Supplementary Figures and legends**


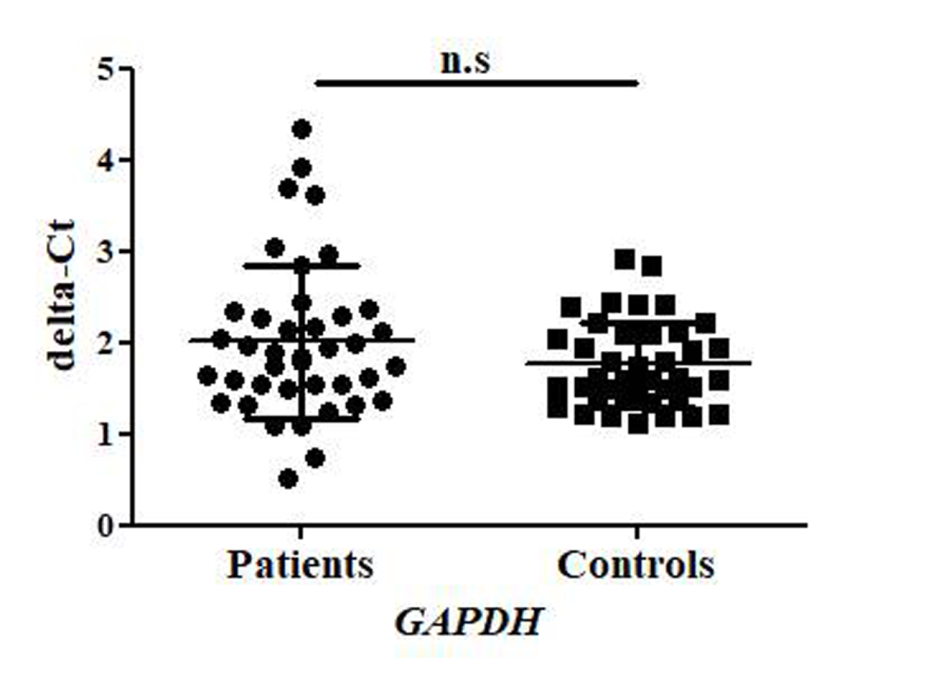


**Figure S1:** Scatterplot showing individual delta-Ct values for Patients and controls.


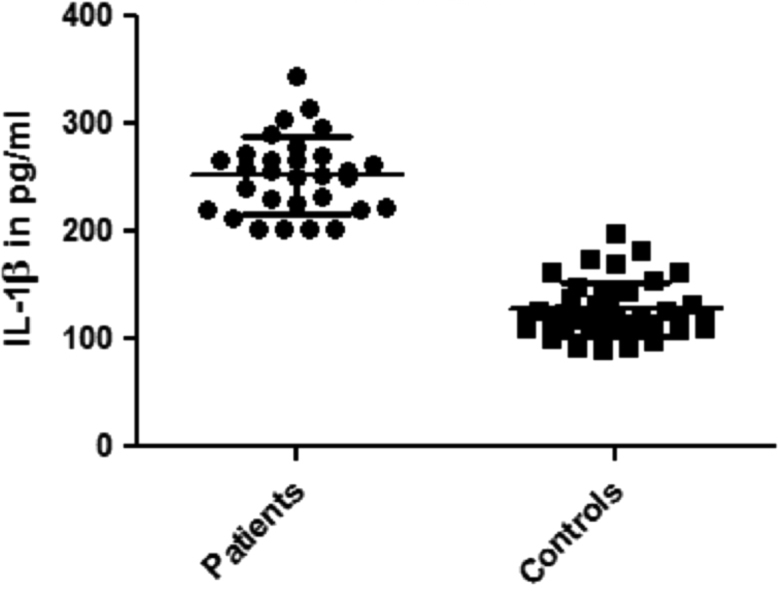


**Figure S2:** Cytokine IL-1β levels in UPEC infected UTI patients and Healthy controls. *(Verma et. al. 2018).*


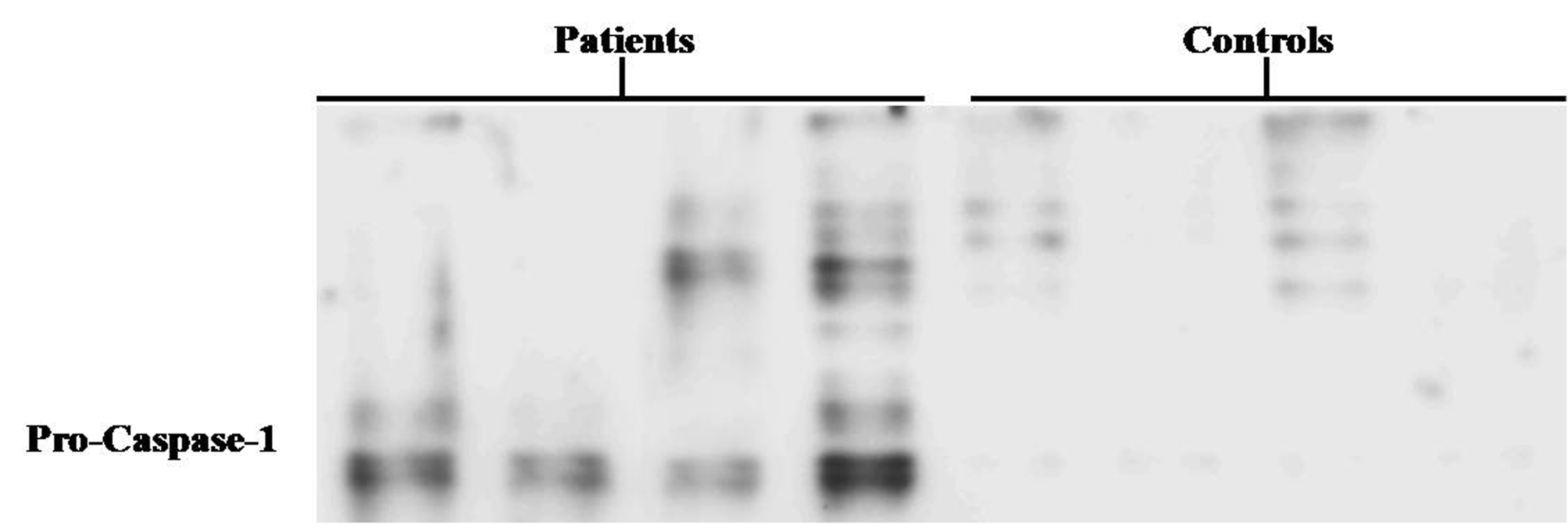


**Figure S3:** Immunoblot showing proCaspase-1 proteins levels in Patient and control group.
